# Subsarcolemmal lipid droplet responses to a combined endurance and strength exercise intervention

**DOI:** 10.14814/phy2.12187

**Published:** 2014-11-20

**Authors:** Yuchuan Li, Sindre Lee, Torgrim Langleite, Frode Norheim, Shirin Pourteymour, Jørgen Jensen, Hans K. Stadheim, Tryggve H. Storås, Svend Davanger, Hanne L. Gulseth, Kåre I. Birkeland, Christian A. Drevon, Torgeir Holen

**Affiliations:** 1 Department of Nutrition, Institute of Basic Medical Science, University of Oslo, Oslo, Norway; 2 Department of Endocrinology, Morbid Obesity and Preventive Medicine, Faculty of Medicine, Oslo University Hospital, University of Oslo, Oslo, Norway; 3 Norwegian School of Sport Sciences, Oslo, Norway; 4 The Intervention Centre, Oslo University Hospital, Oslo, Norway; 5 Department of Anatomy, Institute of Basic Medical Science, University of Oslo, Oslo, Norway

**Keywords:** Electron microscopy, exercise, insulin sensitivity, lipid droplets, lipophagy, muscle

## Abstract

Muscle lipid stores and insulin sensitivity have a recognized association although the mechanism remains unclear. We investigated how a 12‐week supervised combined endurance and strength exercise intervention influenced muscle lipid stores in sedentary overweight dysglycemic subjects and normal weight control subjects (*n* = 18). Muscle lipid stores were measured by magnetic resonance spectroscopy (MRS), electron microscopy (EM) point counting, and direct EM lipid droplet measurements of subsarcolemmal (SS) and intramyofibrillar (IMF) regions, and indirectly, by deep sequencing and real‐time PCR of mRNA of lipid droplet‐associated proteins. Insulin sensitivity and VO_2_max increased significantly in both groups after 12 weeks of training. Muscle lipid stores were reduced according to MRS at baseline before and after the intervention, whereas EM point counting showed no change in LD stores post exercise, indicating a reduction in muscle adipocytes. Large‐scale EM quantification of LD parameters of the subsarcolemmal LD population demonstrated reductions in LD density and LD diameters. Lipid droplet volume in the subsarcolemmal LD population was reduced by ~80%, in both groups, while IMF LD volume was unchanged. Interestingly, the lipid droplet diameter (*n* = 10 958) distribution was skewed, with a lack of small diameter lipid droplets (smaller than ~200 nm), both in the SS and IMF regions. Our results show that the SS LD lipid store was sensitive to training, whereas the dominant IMF LD lipid store was not. Thus, net muscle lipid stores can be an insufficient measure for the effects of training.

## Abstract (precis)

We have investigated the muscle storage lipids responses to exercise, finding that subsarcolemmal lipid droplets are reduced 80%. Interestingly, we find that the lipid droplet diameter distribution was skewed, with a marked lack of lipid droplets smaller than 200 nm.

## Introduction

Increased intramyocellular lipid stores have been proposed to be an early hallmark in the development of type 2 diabetes (T2D) (Perseghin et al. 1999). Whereas strong correlations between insulin resistance and intramyocellular lipid stores (IMCL) have been demonstrated in T2D subjects and in T2D offspring (Pan et al. 1997; Jacob et al. 1999; Perseghin et al. 1999; Levin et al. 2001), the largest cross‐sectional study (*n* = 105) reports no connection between insulin sensitivity and IMCL in normal weight subjects (BMI = 24.6 ± 5) (Thamer et al. 2003).

Although physical activity is known to improve insulin sensitivity, and has been used in the prevention and treatment of T2D for almost 100 years (Goodyear and Kahn 1998), the link between exercise, insulin sensitivity, and intramyocellular lipid stores remains still unclear. There is no clear pattern in 38 long‐term exercise intervention studies measuring intramuscular triacylglycerol (TAG) lipid droplet (LD) stores by biochemical extraction (*n* = 7), electron microscopy (EM) (*n* = 7), magnetic resonance spectrometry (MRS) (*n* = 9), and oil red O (ORO)‐staining histology (*n* = 15) (Morgan et al. 1969; Kiessling et al. 1974; Hoppeler et al. 1985; Howald et al. 1985; Hurley et al. 1986; Kiens et al. 1993; Wang et al. 1993; Suter et al. 1995; Phillips et al. 1996; Bergman et al. 1999; Malenfant et al. 2001b; Gan et al. 2003; Helge and Dela 2003; Schrauwen‐Hinderling et al. 2003a, 2010; Bruce et al. 2004; He et al. 2004; Kim et al. 2004; Pruchnic et al. 2004; Tarnopolsky et al. 2007; De Bock et al. 2008; Dube et al. 2008, 2011; Praet et al. 2008; Solomon et al. 2008; Toledo et al. 2008; Yokoyama et al. 2008; Shah et al. 2009; Ith et al. 2010; Machann et al. 2010; Meex et al. 2010; Nielsen et al. 2010; Haus et al. 2011; Van Proeyen et al. 2011; Bajpeyi et al. 2012; Lee et al. 2012; Shaw et al. 2012; Shepherd et al. 2013).

In particular in obese subjects (body mass index; BMI ≥ 30) the results are very conflicting, with four studies showing a reduction in muscle lipid stores (Bruce et al. 2004; Solomon et al. 2008; Machann et al. 2010; Bajpeyi et al. 2012), six studies showing an increase (Malenfant et al. 2001b; Dube et al. 2008, 2011; Haus et al. 2011; Lee et al. 2012; Shaw et al. 2012), and eight studies showing no effect of exercise (Gan et al. 2003; He et al. 2004; Praet et al. 2008; Toledo et al. 2008; Meex et al. 2010; Nielsen et al. 2010; Bajpeyi et al. 2012; Lee et al. 2012).

At normal weight (BMI ≤ 25), nine studies show enhanced muscle lipid stores after training (Morgan et al. 1969; Kiessling et al. 1974; Hoppeler et al. 1985; Hurley et al. 1986; Kiens et al. 1993; Phillips et al. 1996; Schrauwen‐Hinderling et al. 2003a; Tarnopolsky et al. 2007; Van Proeyen et al. 2011; Shepherd et al. 2013), although some of the results were nonsignificant due to large variation, or could be due to dietary effects. Moreover, five studies reported no or modest reduction of lipid stores (Suter et al. 1995; Kim et al. 2004; Tarnopolsky et al. 2007; De Bock et al. 2008; Bajpeyi et al. 2012). In contrast, in overweight subjects (BMI 25‐30), there are comparatively few studies, with modest effects on LD lipid stores (Bergman et al. 1999; Helge and Dela 2003; Bruce et al. 2004; Pruchnic et al. 2004; Yokoyama et al. 2008; Machann et al. 2010; Meex et al. 2010).

Whereas diet alone in some studies has a substantial effect on muscle lipid stores in lean (Van Proeyen et al. 2011), as well as obese (Goodpaster et al. 2000; Dube et al. 2011) subjects, other studies show no effect (Petersen et al. 2005; Rabol et al. 2009). Furthermore, studies on gastric bypass surgery (Gray et al. 2003; Mingrone et al. 2003) and dietary effects on lipid repletion after exercise (Starling et al. 1997; van Loon et al. 2003b; Zehnder et al. 2006) have shown significant effects on lipid stores. Moreover, muscle lipid stores may increase within 3–4 h due to the increased plasma lipids, as demonstrated in both clamp lipid infusion studies (Bachmann et al. 2001; Boden et al. 2001), and in a study of nonexercising biceps brachii muscle during ergometer cycling (Schrauwen‐Hinderling et al. 2003b).

Indirect studies with isotope labeling demonstrate that utilization of lipid stores in muscle depends on the intensity of the acute exercise (Jansson and Kaijser 1987; Romijn et al. 1993). It has also been shown directly that intensity of exercise has differential effect on lipid depletion in endurance runners during workloads of 69%, 74%, and 84% of VO_2_max (Brechtel et al. 2001) and in a study with 55% VO_2_max continuous cycling versus intermittent cycling at high intensity (Essen et al. 1977).

Acute exercise studies, in contrast to long‐term exercise studies, clearly demonstrate depletion of muscle lipid stores. Using lipid extraction (*n* = 11), EM (*n* = 3), MRS (*n* = 12), and ORO‐staining histology techniques (*n* = 2), most studies show depletion of muscle lipid stores during exercise (Froberg and Mossfeldt 1971; Oberholzer et al. 1976; Lithell et al. 1979; Jansson and Kaijser 1987; Staron et al. 1989; Essen‐Gustavsson and Tesch 1990; Wendling et al. 1996; Starling et al. 1997; Kiens and Richter 1998; Rico‐Sanz et al. 1998, 2000; Boesch et al. 1999; Krssak et al. 2000; Brechtel et al. 2001; Decombaz et al. 2001; Sacchetti et al. 2002; Steffensen et al. 2002; Watt et al. 2002; Johnson et al. 2003; van Loon et al. 2003a,b; Schrauwen‐Hinderling et al. 2003b; McInerney et al. 2005; Koopman et al. 2006; Roepstorff et al. 2006; Zehnder et al. 2006; Jenni et al. 2008; Egger et al. 2013). Despite the technical difficulties of measuring intramuscular lipid stores (Wendling et al. 1996; Howald et al. 2002; De Bock et al. 2007), very few studies apply more than a single measurement technique.

Previous studies have indicated that LD in skeletal muscles are located in two subpopulations, the subsarcolemmal (SS) region and the intermyofibrillar (IMF) region (Malenfant et al. 2001a,b; He et al. 2004; Nielsen et al. 2010; Jonkers et al. 2012). SS LD metabolites might have local effects on insulin sensitivity, due to the proximity to the muscle fiber nuclei and signaling pathways of the sarcolemma, perhaps via DAG, ceramides, or other lipid metabolites (Coen and Goodpaster 2012).

In light of the unclear effects of long‐term exercise on muscle lipid stores, we hypothesized that exercise affects muscle fiber regions and the local LD parameters differently. The aim of this study was to perform a large‐scale, direct EM characterization of LD parameters in the SS and IMF muscle fiber regions. Our data show that the SS LD population is strongly reduced, whereas the IMF LD population was unchanged both in the normal weight control group (BMI = 23.7) and in the overweight dysglycemic group (BMI = 28.5). This suggests that future studies should take regional effects into account when measuring muscle fiber lipid stores. Furthermore, the absence of small lipid droplets and the presence of lipoautolysosomes indicate that the metabolic mechanism of LD turnover is incompletely understood.

## Materials and Methods

### Study design

Details of the whole MyoGlu study are being published elsewhere by (T.M. Langleite, J. Jensen, F. Norheim, H.L. Gulseth, D. Tangen, K.J. Kolnes, A. Heck, T. Storås, G. Grøthe, M.A. Dahl, A. Kielland, T. Holen, H.K. Stadheim, A. Bjørnerud, E.I. Johansen, B. Nellemann, K.I. Birkeland, C.A. Drevon, unpubl. ms.).

## Subjects

Eighteen sedentary, middle‐aged male subjects provided samples for the electron microscopy study of muscle lipid stores. Participants in the dysglycemic group (*n* = 8) and the control group (*n* = 10) differed mainly in BMI (28.5 and 23.7, respectively), body fat percentage and in glucose tolerance status (Table 1).

**Table 1.** Subject characteristics.

	Control group		Dysglycemic group	
	Pre training	Post training	Pre training	Post training
*n*	10		8	
Age (years)	51.4 ± 7.1	–	53.0 ± 5.6	–
BMI (kg/m^2^)	23.7 ± 2.0	23.6 ± 1.9	28.5 ± 2.1	27.4 ± 1.8
Body fat (%)	18.2 ± 3.5	17.5 ± 2.7	24.5 ± 5.6	24.1 ± 5.1
FFM (kg)	64.4 ± 6.1	65.0 ± 6.1	66.4 ± 5.0	66.2 ± 5.2
GIR (mg/kg/min)	7.9 ± 1.6	10.7 ± 2.9	4.3 ± 2.1	5.6 ± 2.2
V0_2_max (mL/kg/min)	43.0 ± 3.0	48.9 ± 5.5	38.2 ± 4.9	43.7 ± 4.2

Data are means ± SD.

### Training program

The participants underwent a 12‐week training period with two 45‐min full‐body strength training sessions and two 45‐min ergometer cycle interval sessions weekly, under supervision. The strength training sessions included a 10‐min aerobic warm‐up and three sets of each of the following exercises: leg press, leg curl, chest press, cable pull‐down, shoulder press, seated rowing, abdominal crunches, and back extension. The endurance training sessions included one session of 7‐min intervals at 85% of maximum heart rate (HR_max_), and one session of 2‐min intervals at > 90% of HR_max_. Compliance did not differ between groups, with an attendance rate of 86% and 88% for the dysglycemic and the control group, respectively.

### Diet

Subjects were asked to stay on their regular diet during the study. Dietary intakes were registered by a food frequency questionnaire (FFQ) before and after the intervention (Johansson et al. 1997). A carbohydrate‐rich meal was provided 90–120 min before the test including bread, cheese, jam, and apple juice, providing 23% of estimated total daily energy expenditure (TEE), on average 2475 KJ. Tests were typically performed in the morning, so the standardized meal was the only intake after the overnight fast.

### Test protocols

Before and after the 12‐week intervention, subjects performed 45‐min ergometer cycle tests at 70% VO_2_max, preceded and followed by muscle biopsies, adipose tissue biopsies, and blood sampling. Participants had a standardized endurance session 3 days before, and then refrained from strenuous physical activity until the test. Other tests before and after the 12‐week intervention included euglycemic hyperinsulinemic clamp to measure glucose infusion rate (GIR), maximum strength, blood pressure, food‐frequency questionnaire (FFQ), waist–hip circumference, and body composition both by bio‐impedance and magnetic resonance imaging (MRI) and MRS.

### Muscle biopsies

Muscle tissue from *m. vastus lateralis* was obtained by Bergstrom needle biopsies (Bergstrom 1962). Three muscle biopsies were collected in connection with the 45‐min bicycle tests at 70% of VO_2_max before (A_1_, B_1_ biopsies), directly after (A_2_, B_2_ biopsies), and after 2‐h rest (A_3_, B_3_ biopsies), before as well as after the 12‐week training period (A‐biopsies before intervention, B‐biopsies after intervention). Muscle biopsies were quickly rinsed in cold PBS and dissected on a PBS‐soaked paper on a cold aluminum plate under a stereo magnifier to remove visible fat, blood, and connective tissue. Tissue for RNA isolation was immediately transferred to RNA‐later (Qiagen) overnight, the solution was then drained off before storing the tissue at −80°C.

### Electron microscopy

Selected bundles of postexercise (A_2_ & B_2_) muscle biopsies for electron microscopy were submerged in cold (4°C) fixative (2% formaldehyde, 2% glutaraldehyde in 0.1 M phosphate buffer (NaPi), pH 7.4) and stored at 4°C for minimum 2 h (maximum 6.5 h) before osmication. The muscle samples were subdivided into five parallels, ≤1 mm pieces and embedded, cut, and contrasted using standard protocols (Holen 2011). Briefly, the samples were rinsed three times in 0.1 M NaPi and placed in 1% osmium OsO_4_ in 0.1 M NaPi for 30–35 min with continual rotation motion. Osmicated samples were stored for ≤ 7 days at 4°C until Durcupan embedding. After three washes in NaPi, a gradient dehydration procedure using 15‐min exposures to 50, 70, 80, and 96% ethanol was performed. Thereafter, three incubations of 20 min in 100% ethanol and two incubations of 5 min in propylene oxide removed last traces of water, before samples were put into Durcupan (Fluka, Sigma‐Aldrich Chemie GmbH, Steinheim, Switzerland) at 56°C for 30 min. The Durcupan mixture was then replaced and samples left overnight at room temperature. Finally, five parallel samples were put into Durcupan capsules per subject time point, and left to polymerize at 56°C for 48 h.

Semithin sections (0.5 *μ*m) from a total of 180 blocks were compared using toluidine blue staining, and the region of best structure chosen for ultrastructural studies. Ultrathin sections of 60 nm were cut using an ultramicrotome from Leica (Vienna, Austria), before being contrasted with 10 mg/mL uranyl acetate (1 min) and 3 mg/mL lead citrate (1 min). Sections were stabilized using carbon‐coated formvar films (Rowley and Moran 1975). Images were obtained using a Tecnai G2 electron microscope from FEI (Hillsboro, OR).

### Lipid droplet volume fraction by point counting

For EM pictures point counting, images were taken under 6000‐fold magnification with size 2048 x 2048 pixels. To make sure the images were randomly chosen and evenly distributed, the position of each window was decided beforehand with a lattice on each section overview.

In order to calculate the volume fraction of LDs in the whole muscle fibers, we designed a lattice system intended to contain at least one LD hit point in each image. As LDs make up less than 2–5% of the total cell volume, with a diameter of ~500 nm (equal to about 58.5 pixels) (Weibel 1969), we set the lattice line separation to 58.5 pixels. This resulted in a 35 x 35 lattice system giving 1225 points in total to quantify the LD volume fraction in the myocytes.

Forty images from each section were selected and uploaded to the database of Science Linker B000 and analyzed by the Image Analyzer software. The hit points of LDs were marked manually on blinded images, and counted by the software. The volume fractions were estimated by the ratio of hit points/total points. In total, 1440 images were analyzed by point counting.

### Lipid droplet density and size distribution in subpopulations

For the EM LD parameters study, SS and IMF regions were randomly chosen and images were taken with 4200‐fold magnification. Twenty SS and 20 IMF images from each block were uploaded to Science Linker B000 and analyzed by the Image Analyzer software. In total, 1440 images, different from the point‐counting images, were used for direct LD parameter analysis.

Each LD structure was manually identified and marked. The diameters were measured from a subfraction of randomly selected LDs in each picture. The software calculated the LD numbers and diameters. From the SS images, the boundaries of SS regions were marked manually and the software calculated the SS area sizes. The LD density was calculated as total LD number divided by SS area size.

The observed LD diameters were corrected for observational bias. A given sphere, sectioned randomly, will result in an observed diameter *d*, whereas the theoretical real diameter *D* is given by *D = 4d/π* (Weibel 1969), which is a bias factor of 1.27. Computer‐assisted numerical analysis supported the theoretical value, with the average section found to be 0.786, that is, a bias factor of 1.27. All our reported LD diameters data are corrected. We have made no effort to correct for minor shrinkage effects of fixation and epoxy embedding. Thus, the reported LD diameters are minimum estimates.

### Quantification of subsarcolemmal space fraction

A total of 140 fibers were randomly selected for muscle fiber width measurement and SS area measurements from 14 blocks of muscle biopsies. 130 × and 4200 × magnification EM pictures were taken from ultrathin sections. SS areas at both sides of each fiber were measured using direct tracing methods at 4200 × magnification. The width of each fiber was measured in triplicate on the 130 × pictures at the same location of which the two 4200 × SS area pictures were taken. The SS area percentage was calculated as the ratio of SS area divided by total muscle section area at each cross section.

### Tissue RNA isolation and cDNA synthesis

Frozen human muscle biopsy pieces were cooled in liquid nitrogen, and crushed to powder in liquid nitrogen‐cooled mortar and pestle. Muscle tissue powder was then poured into 1 mL QIAzol Lysis Reagent (Qiagen), and homogenized using TissueRuptor (Qiagen) at full speed for 15 sec twice. Total RNA was isolated from the homogenate using miRNeasy Mini Kit (Qiagen). RNA integrity and concentration were determined using Agilent RNA 6000 Nano Chips on a Bioanalyzer 2100 (Agilent Technologies Inc). Using High‐Capacity cDNA Reverse Transcription Kit (Applied Biosystems, Foster City CA), 200 ng of totalRNA was converted to cDNA for TaqMan real‐time RT‐PCR.

### TaqMan real‐time RT‐PCR

The cDNA reaction mixture was diluted in water and an cDNA equivalent of 10 or 25 ng RNA input from muscle was analyzed in each sample. Quantitative real‐time RT‐PCR was performed with reagents and instruments from Applied Biosystems in the 96‐well format using a 7900HT Fast instrument and the SDS 2.3 software (Applied Biosystems). Predeveloped primers and probe sets (TaqMan assays, Applied Biosystems) were used to analyze mRNA levels of Perilipin‐1 (*PLIN1*, Hs01106925_m1), *PLIN2 (*Hs00605340_m1), PLIN3 (Hs00998416_m1), *PLIN4* (Hs00287411_m1), *PLIN5* (Hs00965990_m1), and beta‐2 microglobulin (*B2M*, Hs00984230_m1). Relative target mRNA expression levels were calculated as 2^−ΔCt^, normalizing data to endogenous B2M.

### High‐throughput mRNA sequencing

The Illumina High Seq 2000 system, at the Norwegian Sequencing Centre, was used for massive parallel bridge PCR amplification of isolated muscle tissue mRNA converted to cDNA. The cDNA was sonically fragmented and then size selected to 51 base pair long reads before amplification. The library size was, on average, 44.1 million single end reads and was run in eight lanes with multiplexing within each lane.

Subsequent analysis was performed using the Tuxedo pipeline (Trapnell et al. 2012), for alignment to a known reference, the UCSC human genome 19, build 2009 CRCh 37, and the transcriptome annotation. Tophat 2.0.8, with Bowtie 2.1.0, was used with default settings and two mismatches were allowed per uniquely aligned read. Cuffdiff 2.1.1 was used for differential gene expression analysis using pooled dispersion method. Time series analyses were used on within‐group variation and group wise comparison was used for between‐group variation analyses.

### Magnetic resonance spectrometry

All participants underwent a muscle MRS scan as part of a full‐body MRI examination within 3 weeks prior to the start of the training period and a new examination within 2 weeks after the final exercise test. MRI/MRS was performed in the evening, and no strenuous exercise was permitted the same day. Scanning was performed on a 1.5T Philips Achieva MR (Best, The Netherlands) applying the Quadrature Body Coil. The MRI method and results are presented in another paper (T.M. Langleite, J. Jensen, F. Norheim, H.L. Gulseth, D. Tangen, K.J. Kolnes, A. Heck, T. Storås, G. Grøthe, M.A. Dahl, A. Kielland, T. Holen, H.K. Stadheim, A. Bjørnerud, E.I. Johansen, B. Nellemann, K.I. Birkeland, C.A. Drevon, unpubl. ms.).

A single voxel spectroscopy acquisition was performed in the *m. vastus lateralis*. A 15 by 10 by 25 mm^3^ voxel was placed in a homogenous area taking care to avoid any visible fat or fascia. Scan parameters were TR/TE: 3000/31.2 ms bandwidth: 2500 Hz, # samples: 4096, #acquisitions: 64.

Peak fitting was performed using Time‐Domain Quantification of ^1^H Short Echo Time Signals (QUEST) as implemented in the jMRUIv5 software package (Scheidegger et al. 2013). A two peak basis set was created. The water peak was sampled in a water phantom with the applied PRESS sequence and the fat peak was modeled as a single peak using the jMRUI Simulation tool. The peaks were fitted using soft constraints on frequency and damping. Fat fraction *f* was calculated according to Ratiney et al. 2004.



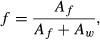



with *A*_*f*_ and *A*_*w*_ denoting the fitted amplitude of the fat and water signals, respectively.

### Statistics

EM images were collected in a blinded, systematic fashion. The blind code was kept by a person not involved in data collection or analysis. The code was broken after the completion of data collection, enabling a third person to perform statistical analysis. Student's *t‐*tests for paired data were used to assess the within‐group variation and *t‐*tests for independent samples to assess between‐group variations.

## Results

The 12‐week training intervention promoted increased VO_2_max and increased insulin sensitivity. VO_2_max increased by 14% both for the dysglycemic group (*P* = 0.002) and control group (*P* = 0.001), respectively. Insulin sensitivity, by glucose infusion rate (GIR), increased by 30 and 35% in the dysglycemic group (*P* = 0.007) and control group (*P* = 0.003), respectively. There was no significant change in BMI during the intervention (*P* = 0.15 dysglycemic group, *P* = 0.54 control group) (Table 1).

### Large‐scale quantitative EM analysis of muscle LD subpopulations

**Figure 1.**
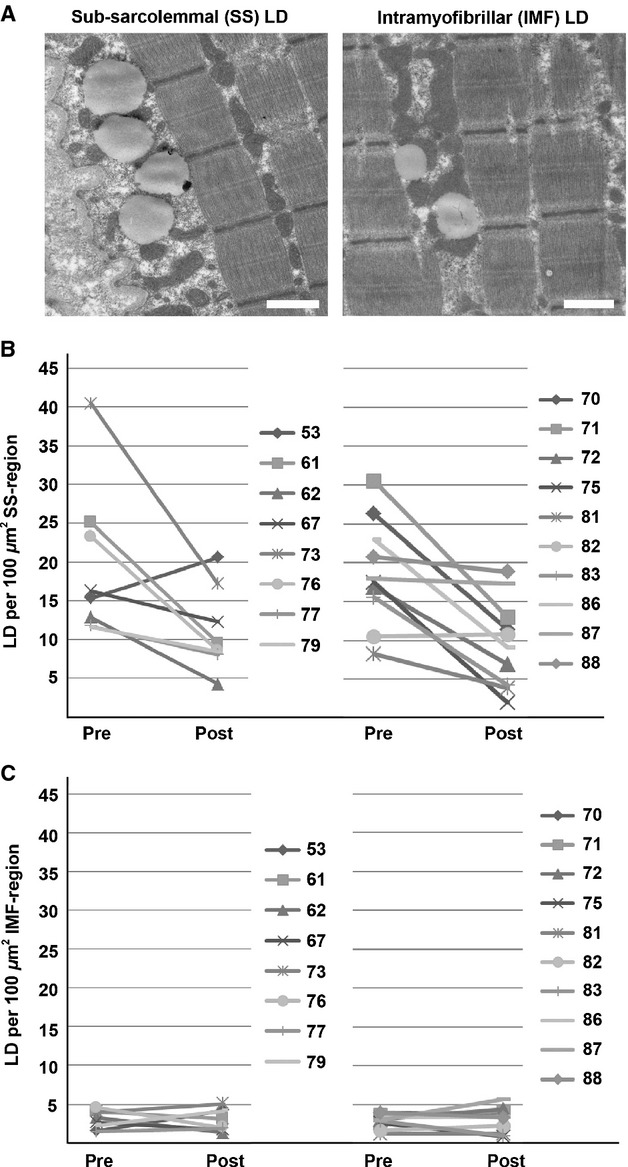
Lipid droplet (LD) numbers in subsarcolemmal (SS) and intramyofibrillar (IMF) regions before and after the 12‐week exercise intervention. (A) Electron micrographs of SS and IMF LD. Scale bar is 1 *μ*m. **(**B) LD density in SS region. Left panel: dysglycemic group subjects. Right panel: control group subjects. (C) LD density in IMF region. Left panel: Dysglycemic group subjects. Right panel: Control group subjects.

In order to study the basic characteristics of muscle LD and the effect of the training intervention, we performed an extensive, blinded, randomized study of 1440 EM images from the SS and IMF regions (Fig. 1A). In total, 14 505 LD were directly counted for density measurements, and of these, 10 958 LD diameters were measured.

The average density of LD in the SS region of 19.6 LD (±9.9 SD) and 18.8 LD (±6.8 SD) per 100 *μ*m^2^ was reduced by 37 and 43% in the dysglycemic group (*P* = 0.03) and the control group (*P* = 0.002), respectively (Fig. 1B).

The LD density of the SS region was about 6‐fold higher than IMF region (Fig. 1C), which had 3.0 LD per 100 *μ*m^2^ (±1.2), and 2.9 LD per 100 *μ*m^2^ (±0.9) in the dysglycemic group and the control group, respectively. The IMF LD density did not change significantly (*P* = 0.93 and *P* = 0.88) during the intervention, with 2.9 LD per 100 *μ*m^2^ (±1.4), and 3.0 LD per 100 *μ*m^2^ (±1.6) in the dysglycemic and the control group, respectively, after the 12‐week training intervention.

Because the IMF region is larger than the SS region, the relative sizes of the SS and IMF LD populations are not directly comparable. We performed a quantification of the relative areas of the SS and IMF regions, observing that the SS area accounts for ~4% of the total muscle area, whereas IMF represents ~96%. Thus, even if the LD density of the SS region is 6‐fold higher than in the IMF region, the total IMF population is approximately 4‐fold larger than the SS LD population.

### LD diameters in the SS and IMF regions and response to training

The average LD diameter in the SS region, corrected for sectional observation bias with a factor of 1.27, was 791 nm (±186 SD) (*n* = 3311), whereas the diameter of LD in the IMF region was 671 nm (±74 SD) (*n* = 7647). Thus, LDs in the SS region were bigger than the LD in the IMF (*P* = 0.008), although with a substantial overlap in size between the two LD populations.

**Figure 2.**
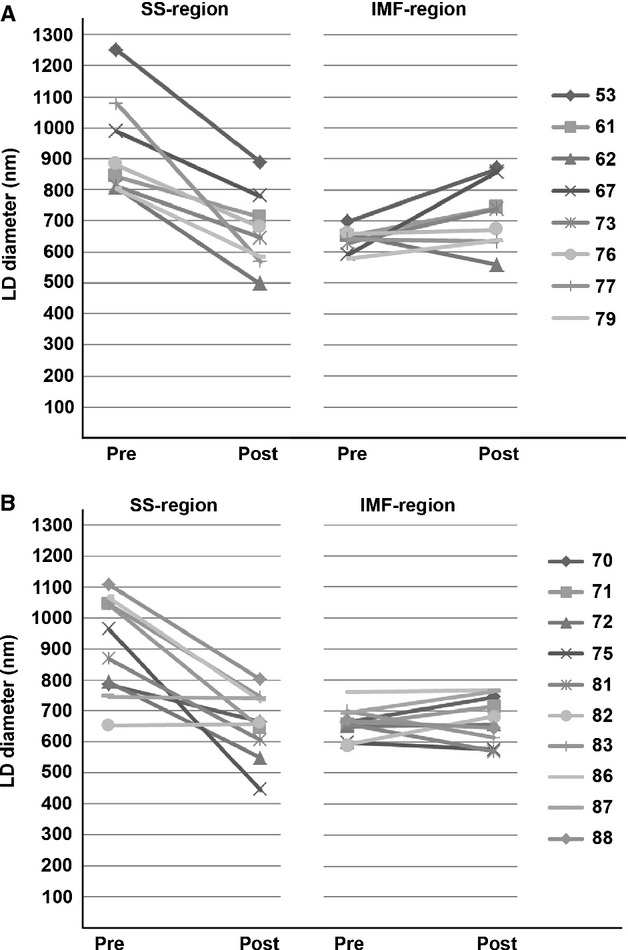
Lipid droplet (LD) diameters in the SS and IMF regions before and the 12‐week training intervention. (A) Dysglycemic group subjects. (B) Control group subjects.

The size of LDs in the two regions responded differently to the training intervention (Fig. 2). The average, corrected diameter of the SS region was 933 nm (±162 SD) and 907 nm (±159 SD) for the dysglycemic group and the control group, respectively. Both groups responded to the training intervention by 28 and 26% reduction in LD diameter (*P* = 0.005 and *P* = 0.001), to 669 nm (±125 SD) and 659 nm (±106 SD), for the dysglycemic and control group, respectively.

The LD diameters in IMF were 638 nm (±39 SD) and 664 nm (±49 SD) before the intervention, and 713 nm (±110 SD) and 674 nm (±73 nm) after the intervention, for the dysglycemic group and the control group, respectively, with no statistically significant change in either group, although the dysglycemic group exhibited a trend toward enhanced IMF LD diameters (*P* = 0.10). Despite this trend, overall we found no significant difference between the dysglycemic group and the control group, on measures of LD density or LD diameter in the SS or IMF regions, neither before nor after 12 weeks of training.

### LD distribution

**Figure 3.**
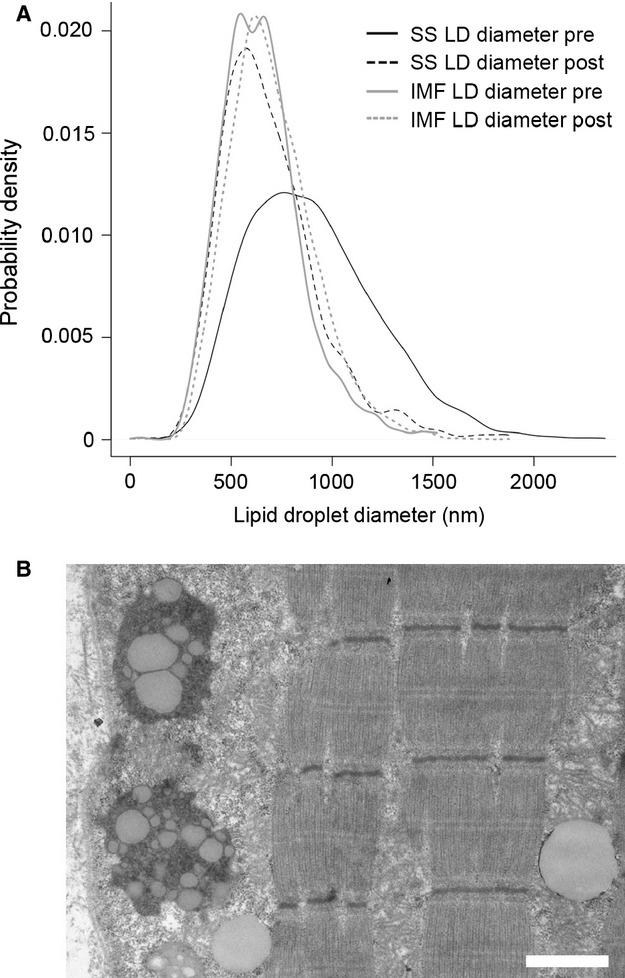
Lipid droplet diameter in SS, IMF, and lipoautolysosomes. (A) Kernel density plot of LD diameter distribution. Distribution is plotted against LD size (horizontal axis) and probability density (vertical axis). SS LD population distribution is shown before (black line) and after (black stippled line) the intervention. IMF LD population distribution is shown before (gray line) and after (gray stippled line) the intervention. (B) Two lipoautolysosomes with multiple internal LD. Smallest LD are ~80 nm. Also present are one SS LD (lower left) and one IMF LD (lower right). Scale bar is 1 *μ*m.

**Figure 4.**
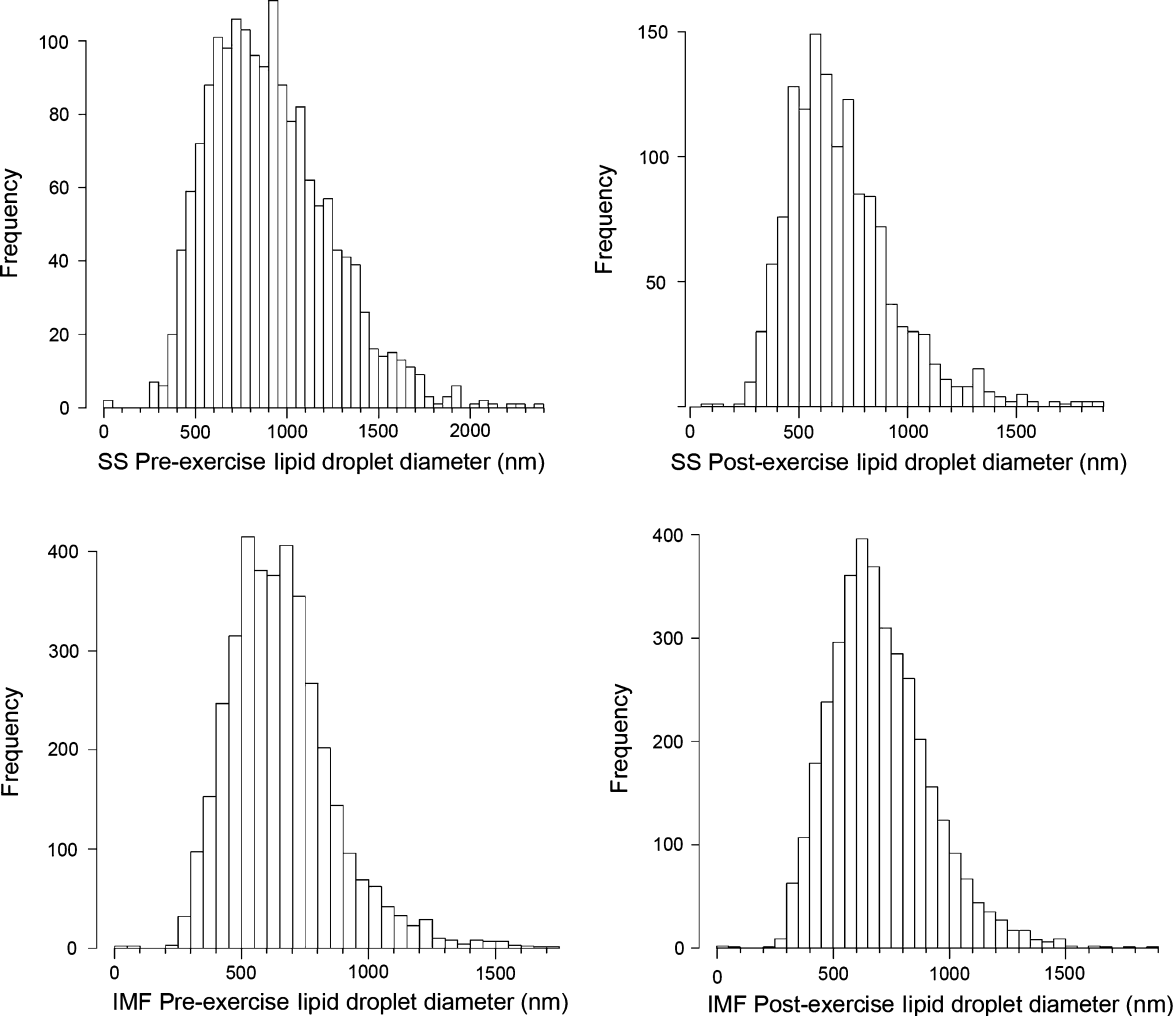
Histograms of lipid droplet size distribution. Lipid droplet numbers are displayed in 50‐nm bins, for SS preexercise, SS postexercise, IMF preexercise, and IMF postexercise diameters.

Analysis of size distribution of LD revealed a much more narrow distribution of the SS LD diameters after the intervention, losing most of the >1000 nm large diameter LD (Fig. 3A, black line SS LD preintervention, black stippled line SS LD post intervention). LD in the IMF region did not change, although a noticeable double peak before the intervention disappeared (Fig. 3A, gray line IMF LD preintervention, gray stippled line IMF LD post intervention). The double peak could also be observed on the 50‐nm window frequency histograms (Fig. 4).

Interestingly, the distribution of all four curves was skewed toward a long tail of large diameter LD, but there were very few LD of diameters lower than ~200 nm (Figs. 3, 4). Whether this phenomenon reflects a lower limit to LD size in muscle in vivo remains currently unclear. Technically, we are able to observe much smaller LD, for example, LD present inside lipoautolysosomes due to lipophagy (Fig. 3B).

### MRS measurement of muscle lipid stores

Total muscle lipids stores, including muscle adipocytes, measured by MRS in *m. vastus lateralis* at baseline before and after the intervention, decreased by 40% (±0.61 SD, *P* = 0.006) for the dysglycemic group and 27% (±0.24 SD, *P* = 0.006) for the control group (Table 2).

**Table 2.** Magnetic resonance spectroscopy of total muscle lipid stores before and after intervention.

Control group				Dysglycemic group			
Subject	Pre training	Post training	Δ (%)	Subject	Pre training	Post training	Δ (%)
#70	0.080	0.094	17	#53	0.025	0.052	107
#71	0.157	0.154	−2.2	#61	0.072	0.037	−48
#72	0.211	0.146	−31	#62	0.113	0.048	−58
#75	0.109	0.038	−65	#67	0.135	0.097	−28
#81	0.057	0.031	−45	#73	0.150	0.045	−70
#82	0.165	0.108	−35	#76	0.144	0.049	−66
#83	0.127	0.114	−11	#77	0.148	0.037	−75
#86	0.061	0.038	−37	#79	0.117	0.025	−79
#87	0.191	0.107	−44				
#88	0.188	0.158	−16				
Average change −27% ± 24 SD					Average change −40% ± 61 SD		

### Point‐counting measurements of muscle lipid stores

Blinded EM point counting of muscle lipid droplet fractional volume (Weibel 1969) showed no significant change in either group when comparing lipid droplets postexercise before and after the intervention (A2 vs. B2 biopsies). The dysglycemic group had average lipid droplet fractional volume of 0.67% (±0.26 SD) at baseline, and 0.63% (±0.39 SD, *P* = 0.84) after intervention. The control group had 0.67% (±0.39 SD) at baseline and 0.67% (±0.46 SD, *P* = 1.0) after intervention.

### Diet

Diet has been reported to affect muscle LD, in particular fat intake (Goodpaster et al. 2000; Dube et al. 2011; Van Proeyen et al. 2011). Dietary intake was monitored in our MyoGlu study, showing no significant change in intake of energy‐providing nutrients during the intervention period. The fat total energy intake was 34.7 and 34.2% before the intervention, and 35.0 and 33.9% after the intervention, for the control group and the prediabetic groups, respectively. Energy intakes from carbohydrates were 43.9 and 41.9% before the intervention, and 43.3 and 42.5% after the intervention, for the control and the dysglycemic groups, respectively. Energy intakes from protein were 15.6 and 17.2% before the intervention, and 15.0 and 17.6% after the intervention, for the control and the dysglycemic groups, respectively. There were minor shifts toward less saturated fat, more fiber and less sugar, despite clear instructions to participants not to change the diet. Such a healthy bias phenomenon has been described previously (Johansson et al. 1997).

### Lipid droplet binding protein mRNA changes

Two recent studies by Shaw, Shepherd, and colleagues correlated two‐to three‐fold changes in LD after exercise training to equally large changes in perilipin PLIN2 and PLIN5 (Shaw et al. 2012; Shepherd et al. 2013). Because the perilipins are correlated with lipid stores (Wolins et al. 2001; Xu et al. 2005; Dalen et al. 2007; Peters et al. 2012), we investigated perilipins mRNA levels in muscle by RNA deep sequencing and TaqMan real‐time RT‐PCR. We found that only PLIN4 was significantly changed in both groups and the whole subject set (*P* < 0.01), which is consistent with no major changes in muscle lipid stores. PLIN1 mRNA, which is almost exclusively expressed in adipocytes, was reduced by ~30% (*P* = 0.10) suggesting a trend toward reduction in muscle adipocytes. Other perilipins did not change significantly (Table 3). RNA sequencing results were validated by TaqMan real‐time RT‐PCR (Table 3).

**Table 3.** *PLIN* mRNA percent changes after a 12‐week training intervention

	All		pT2D		Control	
	mRNAseq	RT‐qPCR	mRNAseq	RT‐qPCR	mRNAseq	RT‐qPCR
PLIN1	−30.4 ± 83.7	−46.5 ± 177.5	−24.9 ± 109.0	−37.3 ± 96.6	−33.1 ± 48.8	−47.6 ± 227.7
PLIN2	−5.8 ± 18.5	−22.9 ± 27.2**	−3.5 ± 16.2	−12.6 ± 15.9	−7.7 ± 21.6	−31.2 ± 32.1*
PLIN3	−6.3 ± 19.3	−16.8 ± 34.0	−2.7 ± 15.3	−3.9 ± 39.7	−9.4 ± 24.0	−27.1 ± 26.3**
PLIN4	−16.2 ± 21.3**	−25.6 ± 26.3**	−15.3 ± 8.1**	−21.3 ± 17.1**	−16.9 ± 31.4*	−29.1 ± 32.4*
PLIN5	14.1 ± 53.4	33.5 ± 88.2	21.6 ± 40.4	37.7 ± 84.8	8.9 ± 68.8	30.2 ± 95.3

All, all subjects (*n *= 18); pT2D, dysglycemic group (*n *= 8); Control, control group (*n *= 10); mRNAseq, RNA deep sequencing data; RT‐qPCR, TaqMan real‐time RT‐PCR. Changes in percent ± SD.

Significant changes indicated by *(*P* < 0.05) and **(*P *< 0.01).

## Discussion

Our 12‐week combined endurance and strength exercise intervention promoted enhanced insulin sensitivity and VO_2_max in both the control and prediabetic subjects; thus, the training intervention was effective.

The lipid droplet volume in the subsarcolemmal LD population was reduced by ~80%, in both groups, while IMF LD were unchanged. Thus, SS LD behaved like ectopic fat and was reduced by exercise. Reduction of the SS lipid droplet population due to exercise has been demonstrated previously in T2D subjects (BMI = 33.5). We extend this observation to overweight, dysglycemic subjects (BMI = 28.5) and normal weight subjects (BMI = 23.7). SS LD metabolites might have local effects on insulin sensitivity, due to the proximity to the muscle fiber nuclei and signaling pathways of the sarcolemma, perhaps via DAG, ceramides, or other lipid metabolites (Coen and Goodpaster 2012).

Interestingly, our large‐scale EM study of lipid droplet diameters (*n* = 10 958) demonstrated a skewed distribution of lipid droplet size, with a lack of small diameter LDs (smaller than ~200 nm), both in the SS and IMF regions, before as well as after the intervention (Figs. 3A, 4). The biological mechanism for this phenomenon remains unclear, but may involve LD fusion, preferential lipolysis of larger LD, limitations of the LD‐coating proteins like perilipins, or lipophagy of small diameter lipid droplets (Singh et al. 2009), as seen in our lipoautolysosomes (Fig. 3B).

Although baseline MRS before and after the training intervention did indicate a reduction in total lipid stores, including muscle adipocytes, measurements of intracellular muscle lipid stores observed by EM point counting, post‐exercise (A_2_?B_2_ biopsies), showed no significant change, which might also suggest reduced LD utilization. A reduction in LD utilization when LD levels are lower, has previously been observed in acute studies of men with lower LD stores than women (Steffensen et al. 2002; Roepstorff et al. 2006). Inversely, higher utilization of LD stores has been observed when LD stores have been increased by high‐fat feeding (Decombaz et al. 2001; Zehnder et al. 2006).

Compliance is a major issue in long‐term interventions. Even in a simple exercise study like MyoGlu, where participants are closely supervised during exercise, and with highly motivated participants instructed not to change their diet, there is a psychological bias toward a more healthy diet (Johansson et al. 1997). Altered diet may affect muscle lipids (Goodpaster et al. 2000; Dube et al. 2011; Van Proeyen et al. 2011); however, our participants had no significant dietary change.

We demonstrate reduced baseline muscle lipid stores after 12 weeks of training, which is consistent with several other training studies (Suter et al. 1995; Bruce et al. 2004; Kim et al. 2004; Solomon et al. 2008; Ith et al. 2010; Machann et al. 2010; Schrauwen‐Hinderling et al. 2010; Van Proeyen et al. 2011).

However, other authors observe increased LD stores, and it remains unclear how chronic exercise influence muscle lipids (Morgan et al. 1969; Kiessling et al. 1974; Hoppeler et al. 1985; Howald et al. 1985; Hurley et al. 1986; Kiens et al. 1993; Wang et al. 1993; Suter et al. 1995; Phillips et al. 1996; Bergman et al. 1999; Malenfant et al. 2001b; Gan et al. 2003; Helge and Dela 2003; Schrauwen‐Hinderling et al. 2003a, 2010; Bruce et al. 2004; He et al. 2004; Kim et al. 2004; Pruchnic et al. 2004; Tarnopolsky et al. 2007; De Bock et al. 2008; Dube et al. 2008, 2011; Praet et al. 2008; Solomon et al. 2008; Toledo et al. 2008; Yokoyama et al. 2008; Shah et al. 2009; Ith et al. 2010; Machann et al. 2010; Meex et al. 2010; Nielsen et al. 2010; Haus et al. 2011; Van Proeyen et al. 2011; Bajpeyi et al. 2012; Lee et al. 2012; Shaw et al. 2012; Shepherd et al. 2013).

The state of confusion might be due to methodological limitations (Wendling et al. 1996; Howald et al. 2002; De Bock et al. 2007), although these limitations have not affected the clear conclusions of studies of the acute effect of exercise on muscle lipids (Froberg and Mossfeldt 1971; Oberholzer et al. 1976; Lithell et al. 1979; Jansson and Kaijser 1987; Staron et al. 1989; Essen‐Gustavsson and Tesch 1990; Wendling et al. 1996; Starling et al. 1997; Kiens and Richter 1998; Rico‐Sanz et al. 1998, 2000; Boesch et al. 1999; Krssak et al. 2000; Brechtel et al. 2001; Decombaz et al. 2001; Sacchetti et al. 2002; Steffensen et al. 2002; Watt et al. 2002; Johnson et al. 2003; van Loon et al. 2003a,b; Schrauwen‐Hinderling et al. 2003b; McInerney et al. 2005; Koopman et al. 2006; Roepstorff et al. 2006; Zehnder et al. 2006; Jenni et al. 2008; Egger et al. 2013). Perhaps the obstacles to obtain representative data on long‐term exercise interventions can be overcome by the use of multiple, validated methods in well‐controlled studies.

The discrepancy in results between acute and chronic exercise may be due to the long time span of a chronic intervention, in which multiple weak factors can influence the lipid stores like obesity/fat percentage, training status, the type, intensity and duration of exercise, muscle fiber type, sex, age, plasma free fatty acids (FFA), leptin/fat store memory fix point, basal fat oxidation, metabolic flexibility, intrinsic lipid droplet (LD) limits, LD subpopulations, glycogen repletion priority, and genetic as well as epigenetic factors.

In our study, SS LD behaved like ectopic fat and was reduced by exercise. In other studies, the IMF LD might behave like a functional local energy store and be increased by exercise. These two factors could cancel out when measuring only net muscle lipid stores. We here show that it is necessary to distinguish between SS and IMF lipid stores. Thus, net muscle lipid stores is an insufficient measure of the effects of training, whereas SS LD seems to be the more sensitive lipid store to training.

Some studies on T2D patients suggest an exercise effect on muscle lipid stores independent of obesity (Goodpaster et al. 2000; Bruce et al. 2004; Bajpeyi et al. 2012), whereas other T2D studies do not (Nielsen et al. 2010; Shaw et al. 2012). It remains unclear whether these differences are caused by insulin resistance or other deficiencies in lipid metabolism. In our study, we observed a correlation (r = 0.41) between LD diameters and insulin sensitivity (GIR) in the dysglycemic group before the intervention. With increased insulin sensitivity, the correlation disappeared after the intervention, which is consistent with the lack of correlation between insulin sensitivity and muscle lipid stores in normal weight subjects (Thamer et al. 2003).

In conclusion, we demonstrate a reduction of total muscle lipid stores in the control and dysglycemic group using MRS, but no corresponding change, postexercise using EM point counting of LD, which may suggest reduced LD utilization with lower LD stores. A large‐scale EM quantification of LD numbers and diameters shows different behavior of two populations of LD. The SS LDs are reduced by ~80%, whereas the intramyofibrillar population of LD did not show any consistent changes. Thus, significant responses to exercise can be masked if only measuring net muscle lipid stores. The large‐scale EM quantification provided a skewed distribution of lipid droplet size, with a lack of small (< 200 nm) diameter LDs both in the SS and IMF regions, before as well as after the intervention, suggesting that LD size regulation mechanisms are insufficiently understood.

## Acknowledgments

The Science Linker B000 database system and Image Analyzer software were kindly provided by professor Niels C. Danbolt, University of Oslo. We thank Ansgar Heck and Birgitte Nellemann for taking biopsies. For assistance in the exercise supervision we thank K.J. Kolnes, D.S. Tangen, T. I Gloppen, T. Dalen, H. Moen, M. A. Dahl, G. Grøthe, E. Johansen, K. A. Krogh, Ø. Skattebo, E. N. Rise. We also thank the Simon Fougner Hartmann Foundation for providing financial support to the YSI glucose analzser at the Diabetes Research Laboratory.

## Conflict of Interest

None declared.
